# Emergent Living Donor Liver Transplantation for Fulminant Hepatic Failure Secondary to Wilson’s Disease

**DOI:** 10.7759/cureus.20653

**Published:** 2021-12-23

**Authors:** Shiva Kumar

**Affiliations:** 1 Gastroenterology and Hepatology, Cleveland Clinic Abu Dhabi, Abu Dhabi, ARE

**Keywords:** fulminant wilson's disease, living donor liver transplantation, fulminant hepatic failure, copper metabolism, heterozygous gene mutation

## Abstract

Emergent living donor liver transplantation in adults with fulminant hepatic failure secondary to Wilson's disease is rarely performed.^ ^We report a case of decompensated Wilson's disease presenting with fulminant hepatic failure treated with an emergent living donor liver transplant. A 25-year-old female presented with fulminant hepatic failure and underwent an emergent living donor liver transplant using a left-lobe graft from her brother. Explant revealed a nodular, cirrhotic liver with numerous yellow-green nodules on the cut surface, and histopathology revealed confluent necrosis and cholestasis with positive copper immunostain. Quantitative hepatic copper was 2119 mcg/g (range: 10−35 mcg/g). Recipient genetic testing revealed c.2930C>T p. (Thr977Met) homozygous variant in the ATP7B gene. The donor was heterozygous for the mutation. The recipient continues to do well three years later with normal ceruloplasmin and urinary copper excretion.

## Introduction

Wilson's disease is an autosomal recessive disorder of copper metabolism caused by a mutation in the ATP7B gene on chromosome 13. It affects one in 30,000 individuals with a reported carrier frequen­cy of one in 90 [1]. Liver transplantation is indicated for patients with Wilson’s disease who present with either fulminant liver failure or end-stage liver disease, and it corrects the underlying hepatic metabolic defect [1]. However, emergent living donor liver transplant in adults with fulminant liver failure due to Wilson's disease is rarely performed [2]. We report a case of decompensated Wilson's disease presenting with fulminant liver failure treated with emergent left-lobe living-related donor liver transplant utilizing a donor that was heterozygous for Wilson's disease mutation. 

This report was presented as an abstract at the Annual Scientific Meeting of the American College of Gastroenterology in October 2019.

## Case presentation

A 25-year-old female with no prior medical history presented with jaundice and fatigue with laboratory studies demonstrating marked hyperbilirubinemia and coagulopathy. Serum ceruloplasmin level was low: 70 mg/L (range: 190-390 mg/L), and 24-hour urine copper was markedly elevated at 7269 μg/24 h (range: 5-50 μg /24 h). The subsequent clinical course was characterized by progressive encephalopathy and coagulopathy, with a model for end-stage liver disease score (MELD) > 40. Initial and subsequent laboratory test results are outlined in Table 1.

**Table 1:** Initial and subsequent laboratory variables The evolution of laboratory variables over the patient's clinical course is shown in this table.

Laboratory Variables	Day 1	Day 4
Total bilirubin (mg/dL)	14.8	36.4
Aspartate aminotransferase (U/L)	165	217
Alanine aminotransferase (U/L)	42	30
Alkaline phosphatase (IU/L)	65	30
Creatinine (mg/dL)	1.0	2.75
International normalized ratio	3.2	5.8

**Figure 1:**
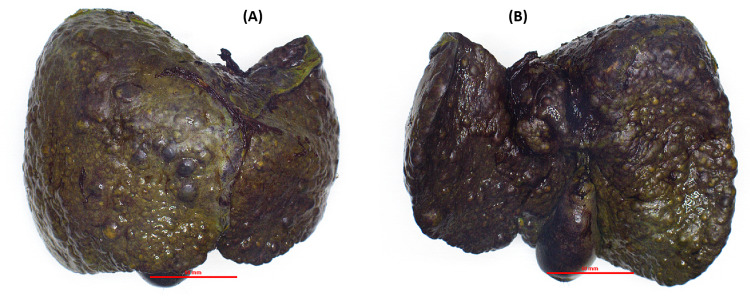
(A and B) Gross examination of the explant liver The explant revealed a nodular, cirrhotic liver.

**Figure 2:**
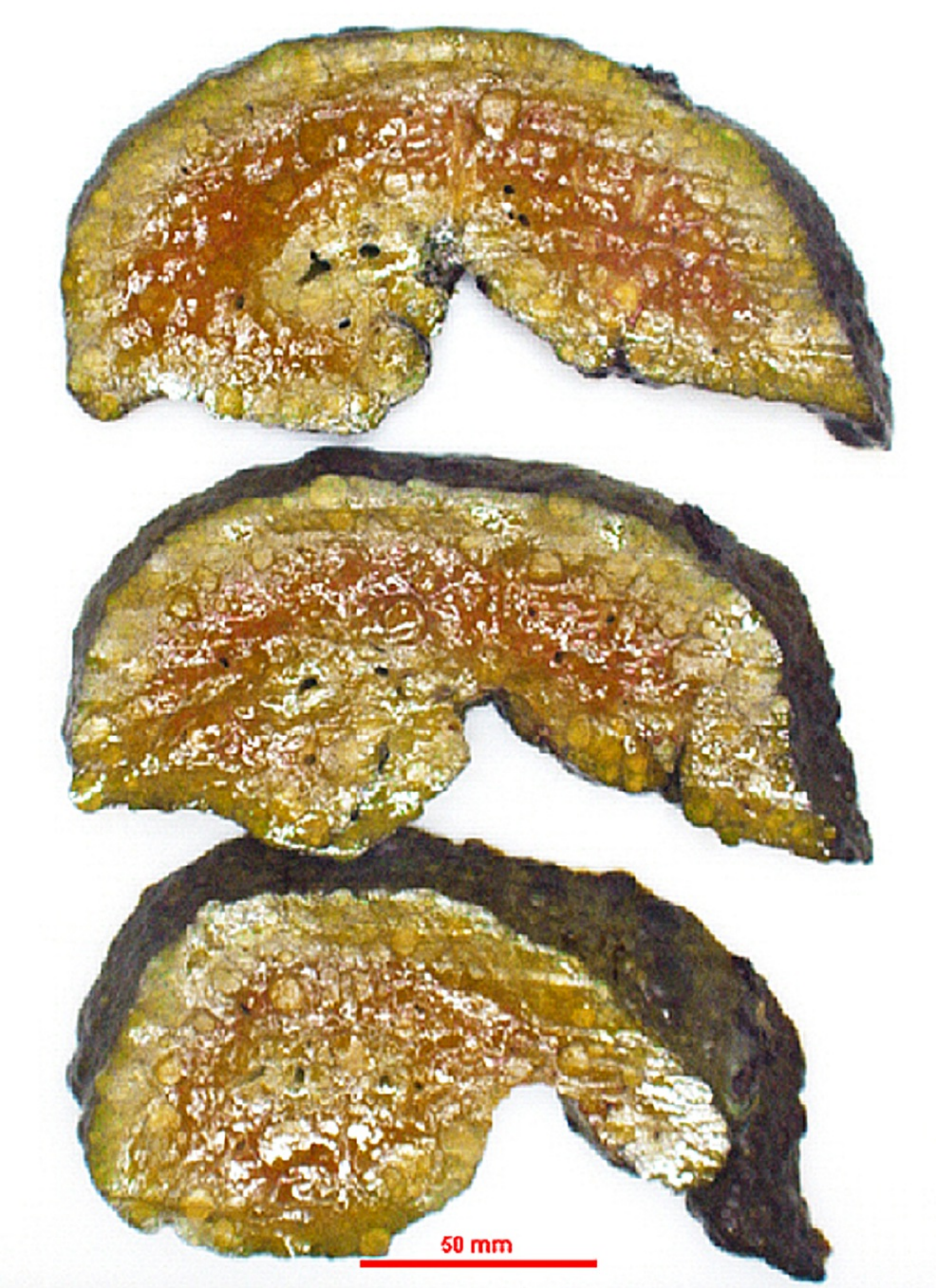
Gross examination of the explant liver with numerous yellow-green nodules on the cut surface

Diagnosed with fulminant hepatic failure due to Wilson's disease, she underwent emergent living donor liver transplantation utilizing a left-lobe graft from her 33-year-old brother, who had normal screening ceruloplasmin. The explant revealed a nodular, cirrhotic liver with numerous yellow-green nodules on the cut surface (Figures 1, 2).

**Figure 3:**
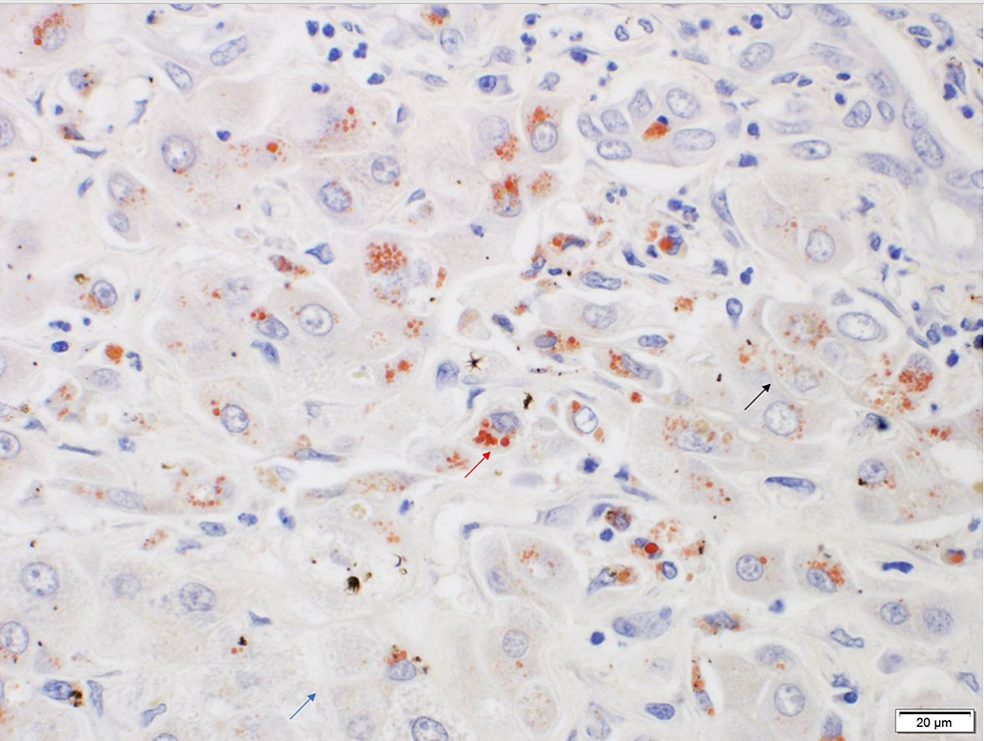
Histopathology showing confluent necrosis (blue arrow) and cholestasis (black arrow) with positive copper immunostain (red arrow)

Histopathology revealed confluent necrosis and cholestasis with positive copper immunostain (Figure 3).

Quantitative hepatic copper content was 2119 mcg/g (range: 10−35 mcg/g). Recipient genetic testing revealed c.2930C>T p. (Thr977Met) variant in homozygosity in the ATP7B gene. Donor was noted to be heterozygous for the mutation. The patient gradually improved and continued to do well 36 months later with excellent allograft function, normal ceruloplasmin, 24-hour urinary copper excretion, and no evidence of neurological manifestations of Wilson's disease.

## Discussion

The introduction of copper-chelating agents such as D-penicillamine and trientine hydrochloride has greatly improved the outcomes of patients with Wilson's disease presenting with chronic liver disease. However, liver transplantation remains the only viable therapeutic option for those presenting with decompensated cirrhosis and fulminant hepatic failure [1].

The overwhelming majority of reported cases of emergent liver transplantation for fulminant hepatic failure secondary to Wilson's disease in adults utilized deceased donor liver transplants, with a few reported cases of living donor liver transplants in children [3]. Given the narrow window of opportunity for transplantation in fulminant hepatic failure and concerns related to heterozygosity in donors, emergent living donor liver transplant is rarely performed in this setting.

However, in countries where deceased organ donation is limited, the only option, given the time-sensitive need for transplantation, is the consideration of emergent living donor liver transplantation. Since a majority of living donor liver transplants are performed using related donors, the possibility of donor heterozygosity remains a concern. In this instance, there was a significant concern for donor heterozygosity since the living donor was the brother of the recipient. 

Encouragingly, in prior reports analyzing recipients with cirrhosis secondary to Wilson's disease, both short-term and long-term outcomes following living donor liver transplant using donors that were heterozygous for the Wilson's disease gene were comparable to those from deceased, unrelated donors [4,5]. Living donor liver transplantation from heterozygous carriers has also been shown to correct the defect in copper metabolism several months after transplantation [6].

## Conclusions

Given the scarcity of deceased donor organs, living donor liver transplantation may be a safe and effective option in decompensated Wilson's disease presenting with fulminant hepatic failure, even if the donor could potentially be heterozygous for the Wilson's disease mutation. Further study is needed to evaluate the long-term impact of potential donor heterozygosity on outcomes following liver transplantation for Wilson's disease.
